# Comparative Analysis of the Dimensional Accuracy and Surface Characteristics of Gears Manufactured Using the 3D Printing (DMLS) Technique from 1.2709 Steel

**DOI:** 10.3390/ma18071461

**Published:** 2025-03-25

**Authors:** Jacek Sawicki, Wojciech Stachurski, Piotr Kuryło, Edward Tertel, Bartłomiej Januszewicz, Emila Brancewicz-Steinmetz, Aleksandra Bednarek

**Affiliations:** 1 Institute of Materials Science and Engineering, Faculty of Mechanical Engineering, Lodz University of Technology, Stefanowskiego 1/15, 90-537 Lodz, Poland; bartlomiej.januszewicz@p.lodz.pl (B.J.); emila.brancewicz-steinmetz@dokt.p.lodz.pl (E.B.-S.); aleksandra.bednarek@dokt.p.lodz.pl (A.B.); 2 Institute of Machine Tools and Production Engineering, Faculty of Mechanical Engineering, Lodz University of Technology, Stefanowskiego 1/15, 90-537 Lodz, Poland; wojciech.stachurski@p.lodz.pl; 3 Institute of Mechanical Engineering, Faculty of Engineering and Technical Sciences, University of Zielona Góra, 65-516 Zielona Góra, Poland; e.tertel@iim.uz.zgora.pl

**Keywords:** gears, post-processing, grinding, metal 3D printing

## Abstract

This article provides a comparative analysis of the dimensional accuracy and post-surface characteristics of gears produced by the 3D printing technique Direct Metal Laser Sintering (DMLS) from 1.2709 steel immediately after printing and after grinding and grinding treatment. The following tests were performed on the fabricated samples: metallography, hardness measurement, self-stress, surface roughness, and the gears’ shape were dimensioned and measured. The results show that post-processing influences the distribution of residual stress and the printed model’s hardness. The results show that heat treatment results in clear directionality marks and micropores, increasing the material’s hardness to 54.3 HRC ± 0.6 HRC, indicating effective strengthening. Grinding significantly improved the holes’ accuracy, changed the compressive intrinsic stresses to a tensile state, and reduced radial runout, improving gear geometries. In addition, it was noted that different results were obtained for roughness parameters depending on the gear tooth tested.

## 1. Introduction

The use of additive manufacturing (AM) technology for metals significantly influences printed components’ dimensional accuracy and surface quality. This impact is highly dependent on the specific characteristics of AM processes, material properties, and processing parameters. Factors affecting the shape dimensions and surface characteristics of metal components manufactured using AM techniques include dimensional accuracy and material shrinkage. Achieving high dimensional accuracy in metal components produced through additive technologies is challenging due to the natural shrinkage and deformations occurring during cooling and solidification. Processes such as selective laser melting (SLM) and electron beam melting (EBM) generate rapid thermal cycles, resulting in residual stresses and deformations that reduce dimensional precision, often necessitating post-processing to align dimensions with the design specification [1,2].

Another factor influencing the shape dimensions and surface characteristics of metal components produced via AM is surface quality and roughness, which are affected by layer thickness, particle size, and scanning speed. Thicker layers and larger particles generally decrease dimensional accuracy and increase surface roughness, creating a textured finish. Roughness can be minimised by controlling layer thickness and optimising laser scanning speed. However, surfaces produced by AM often exhibit a base level of roughness due to the layered process, which may require mechanical finishing in critical applications [3]. Additionally, anisotropy and the microstructural properties of materials are critical, as the layered nature of AM can induce anisotropic characteristics in the printed component. Components constructed layer by layer often show variable properties along different axes due to differences in heat distribution and cooling rates. Anisotropic microstructures, such as elongated grains along the printing direction, can weaken specific mechanical properties and impact shape accuracy by influencing material shrinkage during cooling [4].

The quality and morphology of the powder used in the additive manufacturing of metal components are fundamental to shape dimensions and surface quality. The consistency of metal powders is essential for maintaining dimensional accuracy. High-quality, spherical particles with uniform size distribution provide better packing density, reduce porosity, and improve layer uniformity. Powders produced via gas atomisation are generally preferred for high-precision applications because their spherical shape minimises voids and enhances surface finish [1,5].

When manufacturing three-dimensional metal components using additive technologies, optimising the process concerning energy input to achieve the intended dimensional accuracy is essential. The energy density applied by the laser or electron beam directly affects both dimensional stability and surface integrity. Higher energy inputs can improve layer bonding but may increase the risk of deformation due to higher residual stresses. In contrast, lower energy inputs can weaken bonding, leading to layer separation and poor surface cohesion. Therefore, optimising energy input is crucial to balancing dimensional stability with surface integrity [4,6].

All of the above factors contribute to the dimensional accuracy and surface quality of components produced through additive manufacturing. While post-processing can correct some dimensional deviations and surface roughness, optimising AM process parameters and powder characteristics remains key to enhancing precision and extending the application of metal additive manufacturing technologies. During post-processing, the interaction between heat treatment and grinding is an important consideration, as it significantly impacts the final properties of components made from 1.2709 steel. Heat treatment affects the microstructure and hardness of the material, which, in turn, affects the grinding process, determining its effectiveness and the quality of the resulting surface. Grinding, in turn, can introduce residual stresses and affect the condition of the surface layer, which is essential for the durability and reliability of components [7,8]. Therefore, understanding and controlling these interactions are key to optimising manufacturing processes and ensuring the high quality and durability of components made from steel materials.

In laser-based additive manufacturing processes, such as laser powder bed fusion methods (L-PBF), defects can significantly impair the mechanical properties of produced components. The most impactful defects include gas porosity, voids, and cracks, which primarily result from rapid thermal cycles of heating and cooling and suboptimal process parameters, such as laser power, scan speed, and layer thickness [9]. For instance, excessive laser power or low scanning speeds can cause keyhole porosity due to trapped vapour, whereas high scan speeds can reduce alloy stability, leading to a lack of fusion defects that weaken structural integrity. The impact of defects varies according to their morphology and distribution. Irregular, elongated pores resulting from a lack of fusion tend to act as stress concentrators, reducing tensile strength and significantly lowering fatigue resistance under cyclic loading. Studies utilising X-ray tomography have shown that these pores, often located near the surface, are particularly detrimental to fatigue performance due to their tendency to initiate cracks under stress [10,11]. To mitigate such defects, it is essential to optimise parameters with particular attention to balancing input energy and to employ finishing techniques, such as hot isostatic pressing (HIP), which can reduce internal porosity, thereby enhancing the plasticity and fatigue life of parts [11]. Consequently, adjusting laser parameters and scanning speeds in AM methods by optimising laser power (e.g., 364 W for Al6061) reduces sintering intensity and splatter, minimising shape deviations caused by excessive melting [12]. This approach can achieve a more uniform surface and reduce the need for post-processing. It is noteworthy that dimensional conformity can be ensured at the modelling stage. Preliminary shape deviation simulations allow for 3D CAD model adjustments before printing. For example, in the production of cylindrical components, deviation analysis allows for predicting diameter shrinkage of approximately 0.1 mm due to cooling and residual stresses [13].

It is necessary to consider a material allowance to achieve an ideal metal component using 3D printing. Shape deviations, such as oversizing or undersizing, can result from thermal distortion, stair-stepping effects, and residual stresses during cooling, all of which can affect dimensional accuracy and surface quality. For example, studies recommend allowances ranging from 0.5 mm to 1 mm, depending on the complexity of the part and its orientation during printing, to ensure sufficient material for post-processing adjustments [14,15,16]. Furthermore, specific allowances of around 0.25 to 0.5 mm are suggested to compensate for shrinkage and ensure that functional surfaces meet tolerance requirements after finishing [17,18].

Ensuring the precision and durability of complex geometries, such as gear wheels’ involute profile, requires specialised technological adjustments. Studies indicate that to mitigate such problems as warping or inaccuracies that may affect gear meshing and wear, appropriate printing parameters must be selected, including layer height, printing orientation, and fill density [19]. Achieving precision and durability for the involute geometry of gear teeth requires an optimal selection of materials and design parameters, such as profile shift coefficient, which can increase tooth volume by up to 10% with a positive shift of 0.5 [20]. Finite element analysis demonstrates that appropriate material selection, such as high-strength steel, can improve resistance to dynamic loads, reducing deformations and extending component lifespan by up to 15%. Furthermore, metal 3D printing allows for a 30% weight reduction while maintaining full strength, which is crucial for precision-focused, weight-sensitive designs [21]. Studies suggest that to improve load distribution and durability of complex geometries, primary attention should be given to pressure angles and the contact points of components [22]. Advanced design tools, such as B-spline curves, allow for tooth profile optimisation to achieve smoother, more accurate surfaces on 3D-printed gear wheels [23]. In conclusion, subtractive machining remains essential for bringing an additively manufactured component to the dimensions specified at the CAD design stage.

Therefore, in this work, we focused on the comparative analysis of the dimensional accuracy and surface characteristics of gears manufactured using a 3D printing (DMLS) technique and 1.2709 steel subjected to grinding. Maraging steel 1.2709, also known as X3NiCoMoTi 18-9-5, is a high-strength alloy used in advanced engineering applications such as gears, pressure moulds, and cold extrusion components. Its exceptional mechanical properties, such as high strength and impact strength, make it an ideal material for manufacturing precision components. Three-dimensional printing technology, particularly the Direct Metal Laser Sintering (DMLS) method, makes it possible to fabricate geometrically complex parts from 1.2709 steel [24]. However, the process can introduce some challenges, such as residual stresses and the anisotropy of mechanical and surface properties due to the directionality of the application of successive layers of the material. Finishing, including grinding, plays a key role in minimising these undesirable effects. Grinding can reduce surface roughness, which is important for improving tribological properties of gears, such as friction and wear [8]. In addition, the process can reduce the concentration of surface stresses, which translates into an increased fatigue life of components. Although there are studies on the properties of gears made using DMLS, there is a lack of systematic analyses focusing on the post-grinding stress distribution and anisotropic surface properties of these components. Understanding the impact of finishing on these aspects is crucial to optimising the manufacturing process and ensuring the high quality and reliability of gears in engineering applications [25]. Therefore, studies focusing on the comparative analysis of the dimensional accuracy and surface characteristics of gears made using the 3D printing (DMLS) of 1.2709 steel, taking into account the impact of the grinding process, are not only necessary but also innovative. Bearing in mind that all models were printed in the same configuration, the results obtained, therefore, are important, and further studies may be needed for other cases. They can provide valuable information on the optimisation of finishing parameters, leading to the improvement of the performance characteristics of these components and the expansion of their industrial applications.

## 2. Materials and Methods

### 2.1. Gear 3D CAD Model

**Figure 1:**
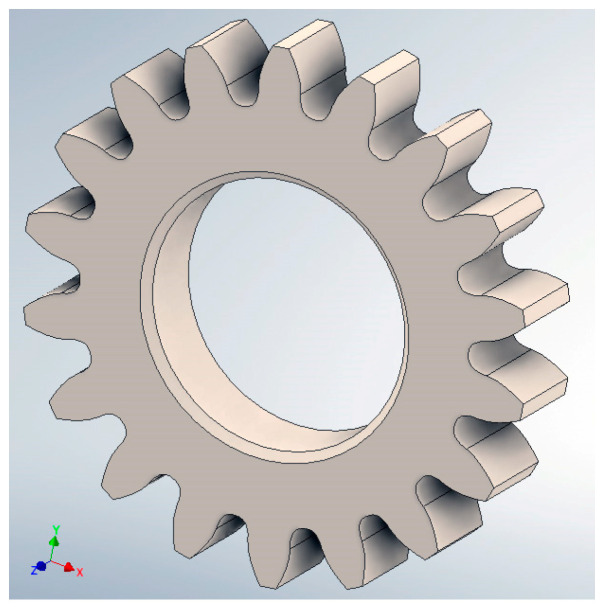
3D CAD model of gear wheel.

A 3D CAD model of the gear wheel was created using Autodesk Inventor Professional 2024 software (Figure 1). The nominal dimension values of the gear wheel listed in Table 1 were used to make the 3D CAD model.

### 2.2. Powder Material

**Figure 2:**
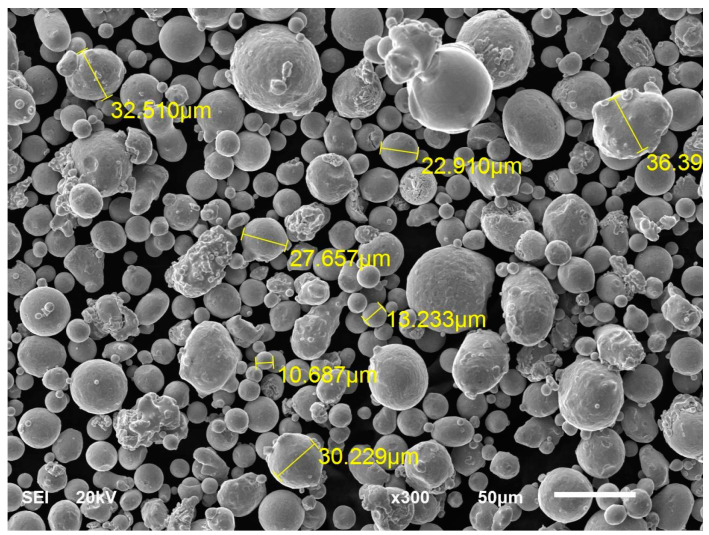
Powder grains used during the implementation of the study SEM.

The input material used for gears was 1.2709 (Maraging steel MS1) tool steel powder (equivalents: American classification: 18Ni-300, German: X3NiCoMoT1 18-9-5). This material is easily heat-treated, which significantly improves hardness and strength properties. Moreover, 1.2709 steel is characterised by reasonable wear and corrosion resistance, as well as very high strength parameters. With these properties, parts made from this steel are used in areas such as automotive, motorsport, aerospace, and tooling. Table 2 presents the chemical composition of 1.2709 by the manufacturer (EOS GmbH, Krailling, Germany). Powder grain size varies and ranges from 10 to 65 μm with a small number of the smallest and largest grains. The predominant fraction is particles with dimensions close to 40 μm (Figure 2). Some authors have studied the consistency of the declared chemical composition and grain size of the powder offered by using EOS, showing good agreement between the parameters of the examined chemical composition and grain size and those declared by the manufacturer [26,27].

The image presented in Figure 2 was captured on a JEOL JSM 6610LV scanning electron microscope (JEOL, Tokyo, Japan). Acquisition parameters were an accelerating voltage of 20 kV, magnification 300 times, and a working distance of 20 mm. The sample was mounted on a microscope holder using double-sided adhesive tape by uniformly spreading the layer of the powder without any pre-treatment and then placed in the microscope chamber and under a high vacuum. The secondary electrons image was then captured.

### 2.3. 3D Printing of Gears

The gear was manufactured from 1.2709 steel powder using the DMLS (Direct Metal Laser Sintering) method. For this purpose, the EOS M290 3D printer (EOS GmbH, Krailling, Germany) was used. During the technological preparation of the printing process, the Materialise Magics program (Materialise, Leuven, Belgium) was used to plan the printing steps and execute SLM printing parameters.

Before the printing process began, the working chamber of the 3D printer was filled with inert gas (nitrogen). The parts were printed on the working platform and preheated to 40°C. Table 3 presents the process parameters used during the printing of the gears.

### 2.4. Post-Processing

**Figure 3:**
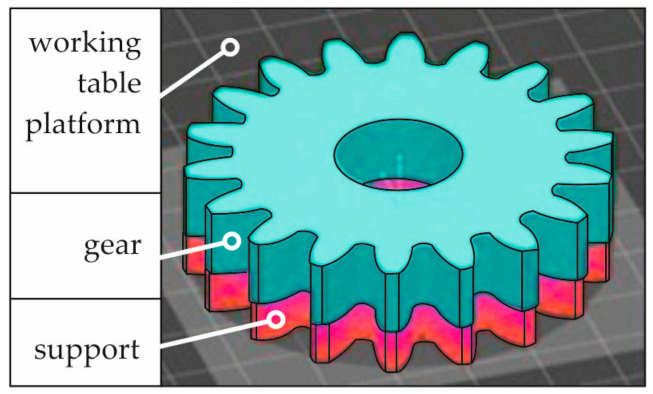
The positioning of the gear on the working table platform during 3D printing.

As shown in Figure 3, the gear was printed on the working table platform in a horizontal position (the gear’s axis of rotation was perpendicular to the surface of the working table), and the printed element was connected to the working table with a support structure. Therefore, post-processing was necessary after printing, involving removing supports and treating some surfaces. For this purpose, the gears were cut off from the working table platform, the support structures were removed, and the surface was cleaned of powder residues. Detaching from the platform and initial removal of the support structures was performed mechanically using hand tools. Next, the surface was finish-machined by grinding. For this purpose, a conventional flat-surface grinder SPD-30B (Jotes Co., Ltd., Łódź, Poland) was used. Grinding was performed using a Vortex-type grinding wheel made of aluminium oxide grains and a ceramic bond. This grinding wheel is designated as IPA60EH20VTX (Norton Saint-Gobein Ltd., Koło, Poland) with dimensions D × T × H = 400 × 50 × 127 mm. The machining conditions suitable for conducting the flat-surface grinding process were determined based on experience from workshop practice and our research, e.g., in [28,29]. A complete set of information is compiled in Table 4.

After machining the end surface of the gear wheel, its central bore was ground. For this purpose, a conventional internal grinder SOH 10 (Jotes Co., Ltd., Łódź, Poland) was used. The grinding was conducted using a grinding wheel made of alumina grains with a vitrified bond, with dimensions D × T × H = 25 × 25 × 10 mm, designated as 99A60KVBE (FTŚ Elephant, Grodzisk Mazowiecki, Poland). The machining conditions suitable for conducting the cylindrical (bore) grinding process were determined based on experience from workshop practice and studies described in the literature, e.g., [30,31]. A complete set of information is compiled in Table 5.

Surface cleaning was performed using a FerroECOBlast 2DV_K1 sandblasting cabin (FerroČrtalič d.o.o., Dolenjske Toplice, Slovenia). The ageing heat treatment was then carried out (Table 4) using a Nabertherm electric induction furnace (Nabertherm, Lilienthal, Germany). The heat treatment parameters are summarised in Table 6.

After heat treatment, the working surface of the gear teeth was ground using the generating gear grinding process, the so-called Niles method. For this purpose, a conventional gear grinder WMW ZSTZ 315 B (WMW Niles, Leipzig, Germany) was used.

**Figure 4:**
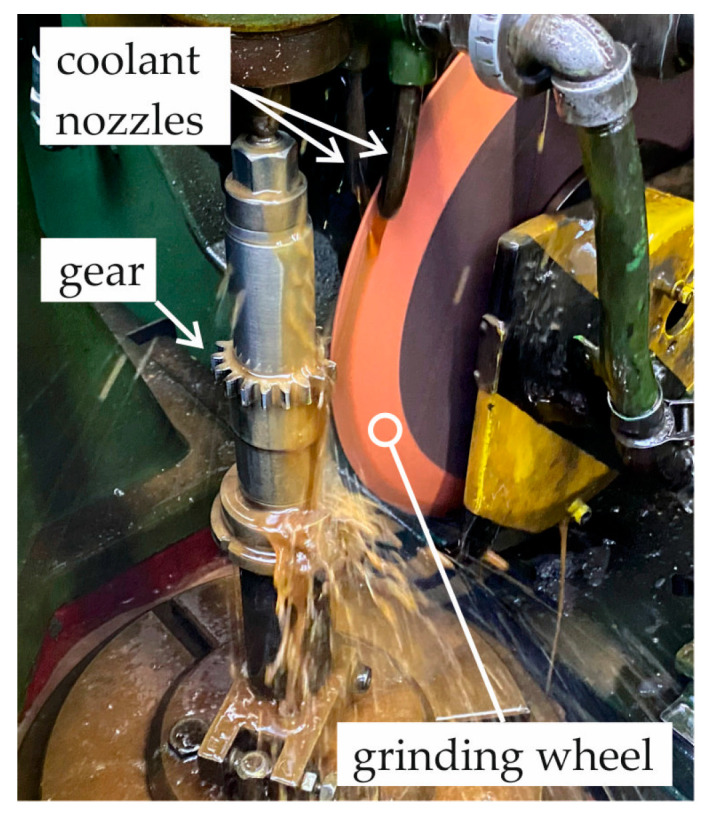
View of workspace during grinding of gear teeth.

An aluminium oxide grinding wheel with a vitrified bond marked 99A80M8V (FTŚ Elephant, Grodzisk Mazowiecki, Poland) was used as the cutting tool. The technical parameters of the grinding wheel are listed in Table 7. Coolant was supplied to the machining zone during grinding using the conventional flood method. Polgrind 1A oil (Naftochem, Kraków, Poland) was used as the grinding fluid, provided by two nozzles at a *Q* = 3 L/min rate. Figure 4 shows a view of the working area and its elements.

The grinding wheel was dressed using a two-single-grain diamond dresser, type M1020, before grinding operations. During grinding, the machining allowance *a_e_* = 0.03 mm was removed on each tooth’s right and left sides (flanks) using a constant grinding speed *v_s_* = 28.3 m/s and workpiece speed *v_w_* = 10 m/min. The grinding parameters used during the tests are listed in Table 8.

The tests were conducted on a machine tool with the kinematic system’s temperature stabilised after half an hour of unloaded operation.

It should be noted that the machining conditions outlined above were selected based on experience gained from years of workshop practice involving the grinding of gear wheels and from our research, the results of which have been published in the literature, e.g., [32,33,34,35]. Regarding the grinding wheel, its specifications (Table 7) indicate that it is suitable for grinding hard steels (>50 HRC), a fact also confirmed by the grinding wheel manufacturer. Combined with the applied machining conditions (Table 8), this allows for obtaining a working gear teeth surface with favourable surface layer properties. The experience above is based on grinding conventionally manufactured steel gear wheels, i.e., non-printed ones. Preliminary grinding trials conducted by the authors did not reveal any differences in executing the machining process or the results obtained.

### 2.5. Metallography

The surface structure and flank face were examined using a Keyence optical microscope (Keyence, Itasca, IL, USA). Observations were made using the High Dynamic Range (HDR) imaging function in a dark field for more detailed observation. Additionally, the optical shadow effect mode enabled the visualisation of surface details. All images were captured at 100× to 500× magnification.

Texture analysis was performed on a JEOL JSM-6610LV microscope with an Oxford Analysis X-MAX80 detector (Oxford, UK) and Aztec v. 4.4 software. The sample was prepared by grinding and polishing using colloidal silica, then placed on the Electron Backscatter Diffraction (EBSD) holder to ensure a 70° tilt.

### 2.6. Hardness Measurements

The hardness of the samples’ surfaces was measured using a KB10BVZ-FA hardness tester (KB Prüftechnik GmbH, Hochdorf-Assenheim, Germany). The hardness was measured using the Rockwell scale according to the standard PN-EN ISO 6508. Three hardness measurements were taken for the sample on metallographic grinds of the wheel tooth. The values were averaged, and standard deviations were determined.

### 2.7. Residual Stress Measurements

Stresses on the ground rings were measured using the X-ray sin2ψ method in ω geometry using a PROTO iXRD device (Proto Manufacturing Ltd., LaSalle, ON, Canada) equipped with two position-sensitive semiconductor detectors. X-rays were generated using a lamp with a Cr anode that emitted characteristic X-rays with a λ wavelength of 2.29 A. A displacement of iron reflex (211) positioned at an angle of 2θ = 156.4° was examined. ½ X-ray elastic constants of S2 = 5/92 1/TPa and S1 = −1.27 1/TPa were used in the calculations. The measurement was taken for an area limited by a collimator with an φ diameter of 2 mm. Exposure time was 1 s. Residual stress measurements were taken for the gear’s face flank, top land, and bottom land. Six measurements were taken of the gear (measurements were taken for every third tooth).

### 2.8. Surface Roughness Measurements

Its roughness was measured to evaluate the quality of the working surface of the gear’s teeth. The measurements were taken twice, directly after printing the gear and then after grinding the teeth using the Niles method. A stationary laboratory contact profilometer, Hommel Tester T8000 (Hommelwerke GmbH, Schwenningen, Germany), and Turbo Wave V7.36 and Hommel Map 4.0 software were used for the measurements.

**Figure 5:**
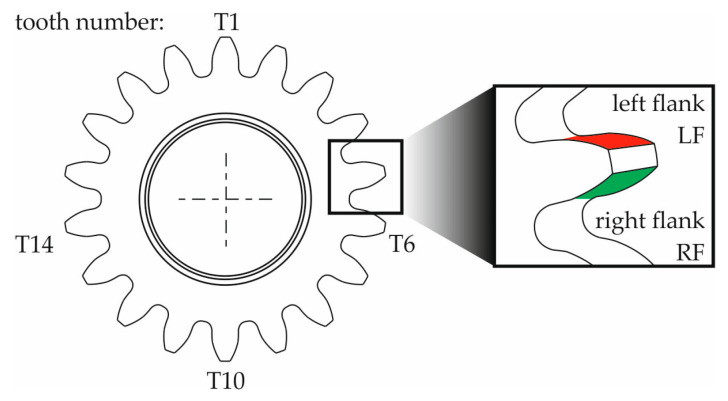
Diagram of the arrangement of the elements of the gear wheel subjected to surface roughness measurements.

Measurements were made on four selected wheel teeth, indicated in Figure 5 and labelled T1, T6, T10, and T14. As shown in Figure 5, the surface roughness was measured on both tooth flanks—the right flank (RF) and the left flank (LF).

The selection of teeth subjected to surface roughness measurement, based on its topography (3D parameters), was preceded by preliminary measurements conducted on all the gear teeth and by determining the *Rz* parameter value (one of the 2D parameters). The comparison of the obtained results did not show significant differences between the individual values of this parameter. Due to this, and considering the overall time consumption of surface topography measurements, measurements were decided based on fewer teeth. Ultimately, roughness measurements were carried out on four teeth, which were also subjected to form and dimensional measurements (radial run-out, tip diameter) using a coordinate measuring machine (described in Section 2.9). The choice of the number of teeth (4) and their placement around the gear circumference at approximately 90° intervals was directly derived from the guidelines related to gear geometry measurements [36].

**Figure 6:**
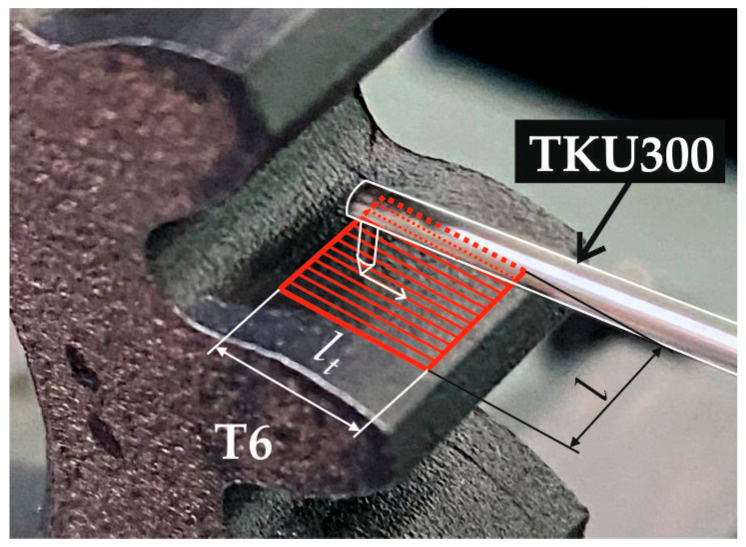
Diagram of the measurement of the topography of the working surface of the tooth of a gear wheel.

The topography of the selected teeth’ working surface was measured during the measurements. For this purpose, the TKU300 measuring stylus was moved perpendicular to the tooth line using 20 parallel passes spread over a width of *l* = 5 mm (Figure 6). Each time, the length of the measuring stylus pass was *lt* = 4.8 mm, and its speed was *v_t_* = 0.05 mm/s. In general, the measurement conditions were selected following PN-EN ISO 3274:2011E and PN-EN ISO 4288:2011E, and their set is summarised in Table 9.

### 2.9. Dimension and Shape Measurements

The measurements of its selected elements, such as the central hole and the teeth, were carried out to assess the dimensional and shape accuracy of the gear. The measurements were taken twice, directly after printing the gear and after post-processing treatments, including grinding the teeth and the hole. The measurements were conducted on a measuring station based on the coordinate measuring machine (CMM) LK V 10.7.6 (LK Metrology LTD, Derby, UK) equipped with a Renishaw SP25M probe along with a measuring stylus ending with a 2 mm diameter measuring ball. The CMM allowed for measurements with a maximum permissible error for length measurement (MPEE) uncertainty, under ISO 10360, of ±0.003 mm. CAMIO and CAMIO Gears (Gear Module v. 1.1.0) software were used to operate the measuring machine.

**Figure 7:**
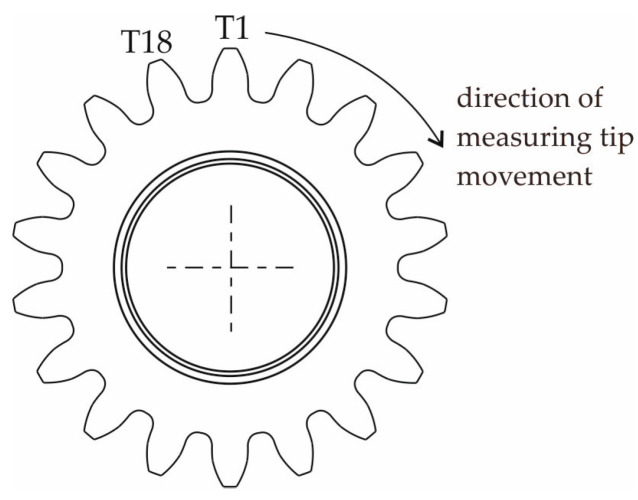
Diagram of the arrangement of the teeth of the gear wheel subjected to measurements.

Measurements were taken from all 18 teeth of the gear wheel. To facilitate the identification of the gear teeth during measurements, one of the teeth was marked as T1, and it was assumed that the subsequent tooth numbers would increase in a clockwise direction (Figure 7).

The measurements of the teeth were used to determine the parameters, enabling the assessment of the dimensional and shape conformity of the 3D CAD model of the gear (Table 1), with the gear printed based on it using 3D printing technology. The following gear parameters were selected for the assessment:

- radial runout *F_r_*,
- tooth thickness *s*,
- tip diameter *d_a_*,
- total pitch error *F_p_*,
- profile slope deviation *f_Hα_*,
- profile form deviation *f_fα_*,
- helix slope deviation *f_Hβ_*,
- helix form deviation *f_fβ_*.

In addition, dimensional and shape accuracy were assessed. The obtained values of selected parameters were compared with the ISO standard, and then, based on this, the accuracy class of the gear tooth design was determined.

**Figure 8:**
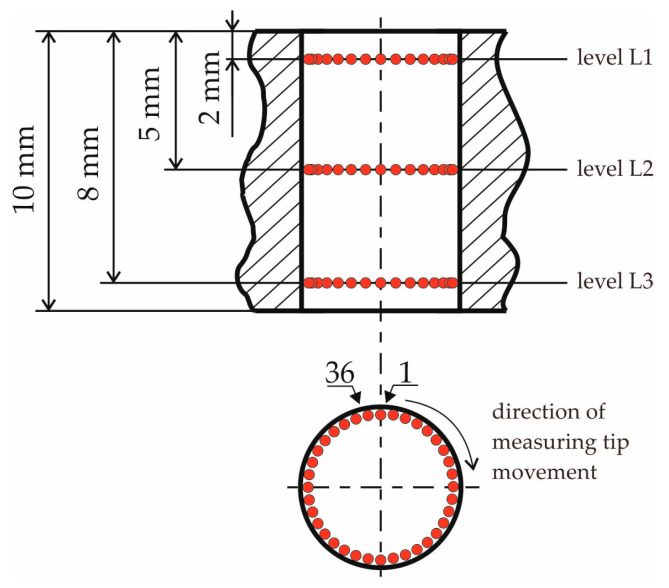
How to arrange the measuring points inside the measured hole.

As shown in Figure 8, measurements of the central hole were taken at three levels (L1, L2, L3) spaced along the hole’s axis. The distance of each level was measured from the top face of the gear wheel and was 2 mm for the L1 level, 5 mm for the L2 level, and 8 mm for the L3 level, respectively. On each of the three levels, 36 measuring points (red dots in Figure 8) were planned, distributed around the circumference of the measuring circle every 10°. The number of measuring points was 36 and corresponded to the total number of teeth and notches of the gear wheel. Three parameters were selected to evaluate the dimensional and shape accuracy of the hole: one related to the dimension of the hole and two related to its shape. These were hole diameter *D*, roundness, and cylindricity (according to PN EN ISO 1101). The first two parameters were determined at three levels (L1, L2, L3).

## 3. Results

### 3.1. Results of Metallography

**Figure 9:**
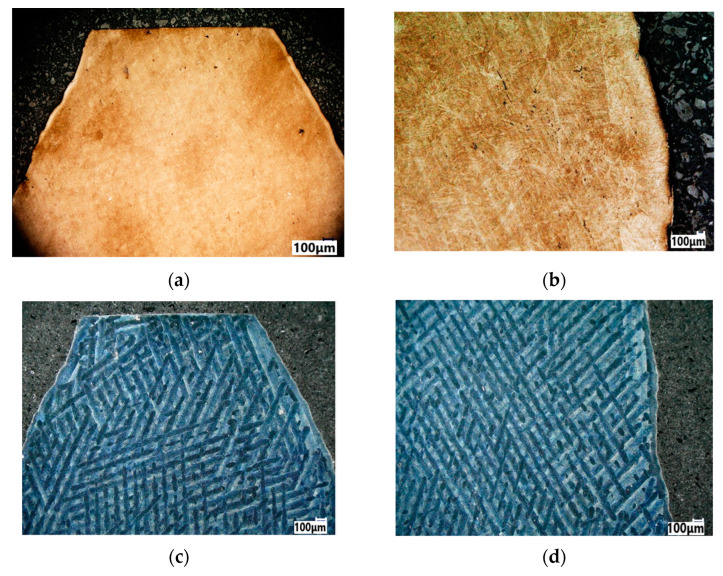
Microstructure of 1.2709 steel: (**a**,**b**) after heat treatment and post-printing structure alignment; (**c**,**d**) the stripes of laser scanning.

Figure 9a,b shows the microstructure of steel alloy 1.2709 after printing and ageing heat treatment. The study of the material’s structure shows the formation of a homogeneous martensitic structure (slate martensite). The heat treatment resulted in a homogeneous and fine-grained structure. Figure 9c,d shows a typical material structure after the laser printing, with visible scanning paths of cellular and columnar morphology. No pores were observed in the matrix.

**Figure 10:**
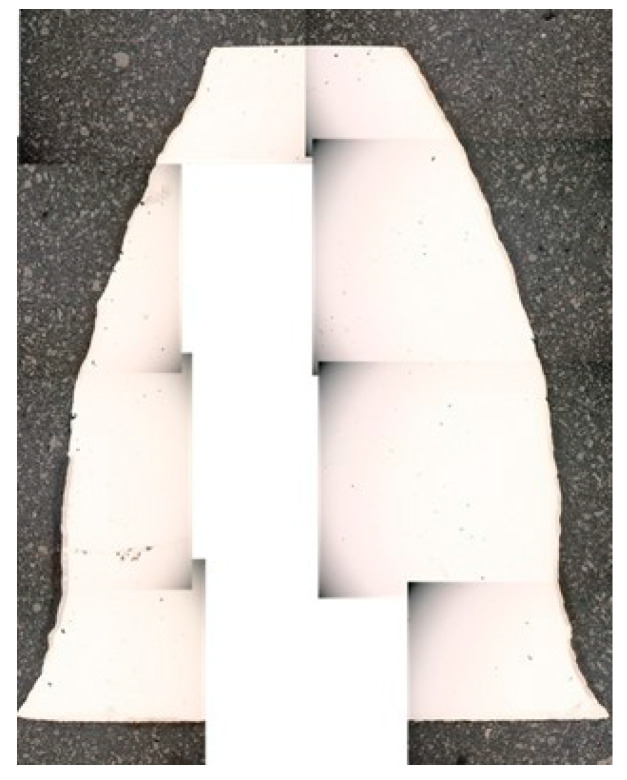
The macroscopic assemblage of a DMLS-printed tooth shape.

Figure 10 shows the assembly of one tooth of the analysed wheel, which shows the quality of the obtained face flanks (evolute). As can be seen from the printing technology, correcting the tooth’s lateral flanks by grinding and adjusting the surface quality to meet the operational requirements is necessary.

**Figure 11:**
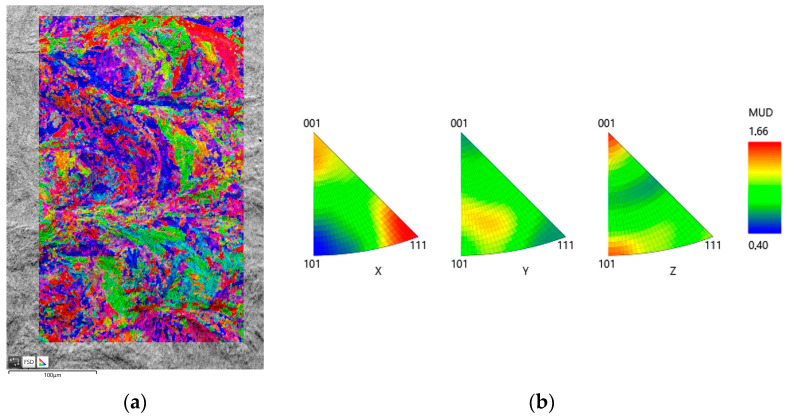
EBSD map of the sample after laser processing: (**a**) IPF X mapping; (**b**) inverse pole figures.

The results of the EBSD analysis are presented in Figure 11. An EBSD analysis was conducted along the building direction (axis X) to verify the grain morphology and examine the crystallographic texture following laser processing. The structure is fine-grained, and the growth of grains during solidification in paths is visible. However, the grains are not elongated or perfectly distributed with the building direction (BD). A possible reason for this deviation is the positioning of the sample inside the SEM chamber, which can vary by a few degrees concerning the vertical and horizontal reference axes. Visually, the orientation of the grains does not possess a preferred crystallographic texture, as depicted by the colours of each grain.

### 3.2. Results of Hardness

The values were averaged, and standard deviations were determined. The wheels’ hardness measurements after supersaturation heat treatment and ageing were characterised by the highest hardness values possible for this alloy steel: 54.3 ± 0.6 HRC, respectively.

### 3.3. Results of Residual Stress

**Figure 12:**
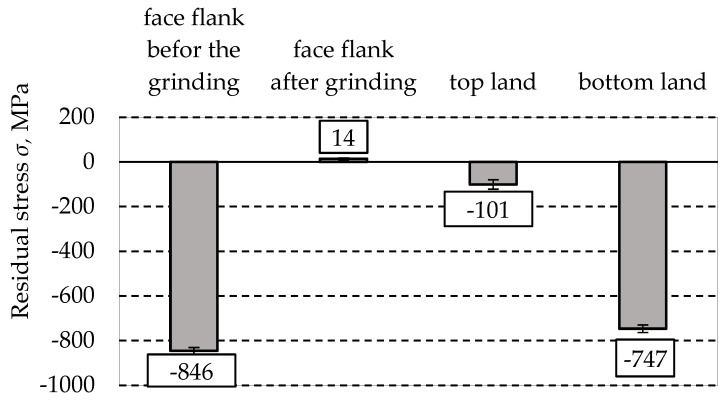
Residual stress measurement results.

Residual measurements revealed the presence of compressive stresses at each of the tested locations (Figure 12). High values of compressive stresses on the face flank and at the bottom land promote an increase in contact fatigue and bending strength. As a result of the superposition of compressive intrinsic stresses with stresses resulting from operating conditions, the appearance of tensile stresses on the surface of hardened elements of friction nodes, which are very unfavourable during operation under contact fatigue conditions, is reduced. The grinding process drastically altered the stress conditions, causing the appearance of tensile stresses.

### 3.4. Results of Surface Roughness Measurements

#### 3.4.1. Parameters of Tooth Working Surface After Printing

**Figure 13:**
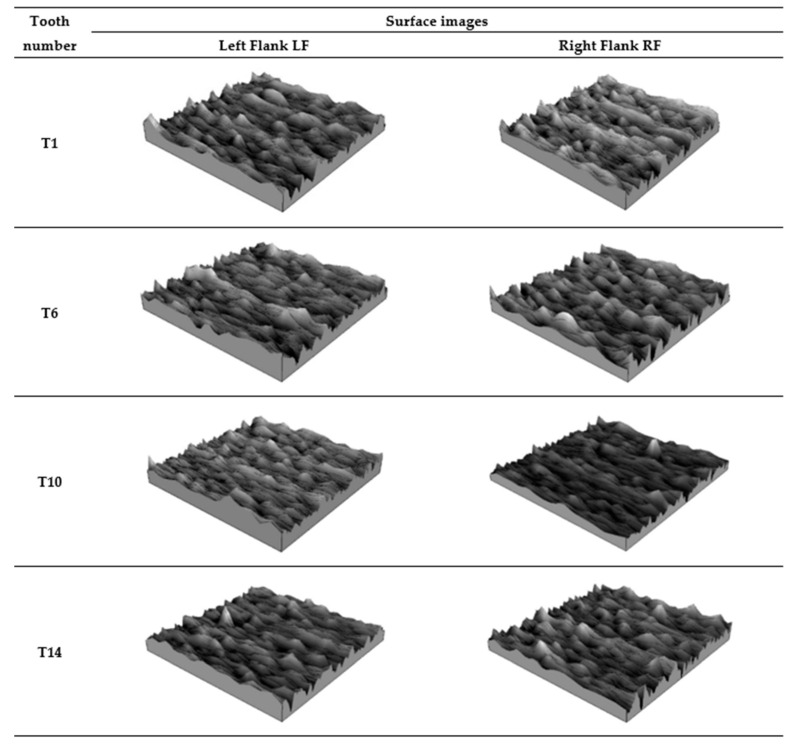
Images of the working surface of the measured teeth—after printing, before grinding.

**Figure 14:**
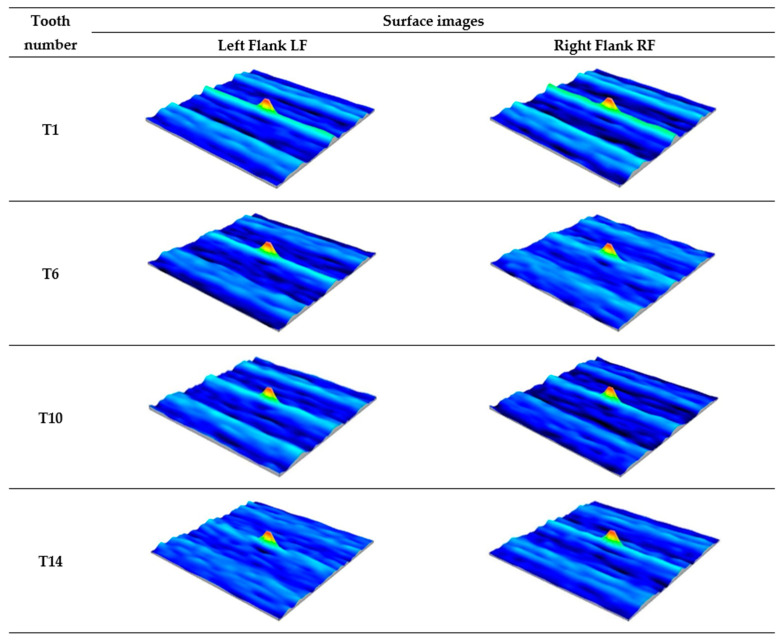
Autocorrelation functions of the working surface of the measured teeth—after printing, before grinding.

Figure 13 summarises the isometric views of the tooth working surface after 3D printing, while Figure 14 shows images of the autocorrelation function of the measured surfaces.

Based on the isometric images of the surfaces presented in Figure 13, it can be preliminarily observed that the working surface of all teeth, both on the left and right flanks, exhibits characteristics of an isotropic surface.

The analysis of the autocorrelation function images of the measured surfaces shown in Figure 14 indicates the presence of a random surface, transitional between anisotropic and isotropic. The photos show signs of slight random periodicity, as evidenced by the unilateral short-wavelength nature of the autocorrelation function decay. It should be noted that actual surfaces often exhibit periodic components of a random nature caused by disturbances in the technological process.

**Figure 15:**
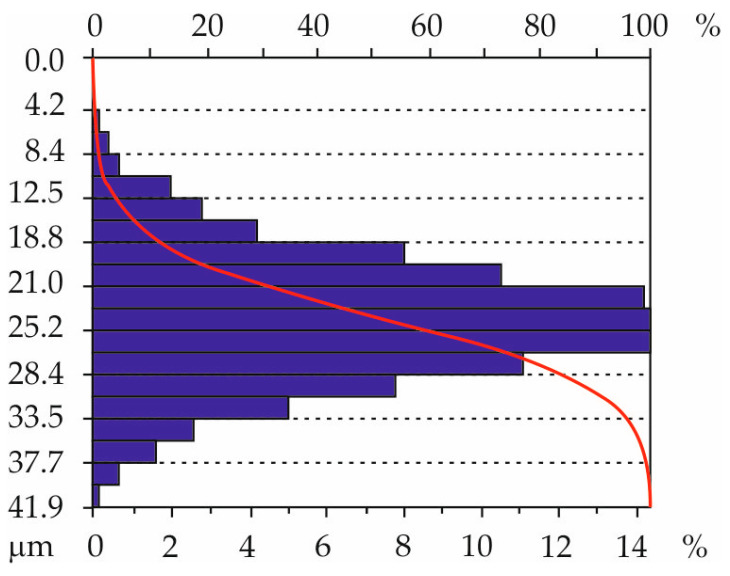
Example distribution of ordinates of the characteristic geometric structure of the surface after printing: tooth T6, right flank RF.

The obtained distributions of the ordinates of the characteristic geometric surface formations confirm the acquisition of random surfaces, as for such surfaces, the ordinate distribution approaches a normal distribution. Figure 15 shows an example graph of one of the surfaces (tooth T6, right flank).

The surface texture index *Str* is the spatial parameter indicating the closer-to-isotropic nature of the examined surfaces. The obtained values are presented in Table 10. As observed, all *Str* parameter values significantly deviate from 0, which is characteristic of isotropic surfaces. The parameter indicating the transitional (between anisotropic and isotropic) nature of the geometric structure of the obtained surfaces is isotropy, the values of which are presented in Table 10. Isotropy is expressed as a percentage, from 0% when the surface is entirely anisotropic to 100% for an utterly isotropic surface.

Table 11 summarises the other values of 3D surface roughness parameters obtained from the measurements. These are amplitude parameters such as *Sz*, *Sa*, *St*, *Sp*, *Sv*, and *Ssk*.

**Figure 16:**
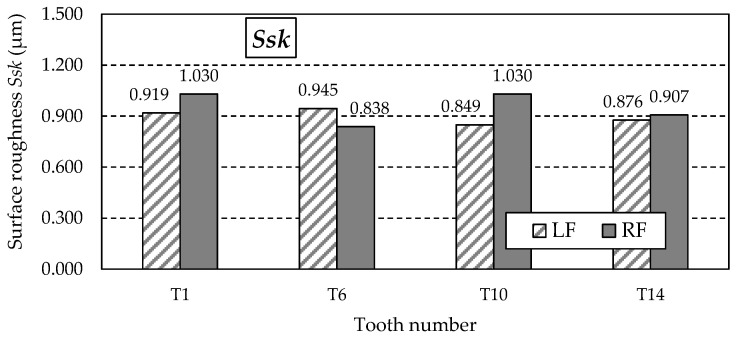
Skewness coefficient *Ssk* for the left flank (LF) and right flank (RF) of the measured teeth.

Figure 16 shows the graph of the *Ssk* parameter values. This parameter measures the skewness of the measured surface, characterising the ordinate distribution’s symmetry relative to the mean plane. The positive *Ssk* parameter values obtained from the measurements indicate surfaces with sharp peaks. It should be noted that skewness is very sensitive to random extreme deviations of the surface in the form of unusual valleys or peaks, which can significantly affect the *Ssk* parameter value while having no impact on the functional properties of the surface.

**Figure 17:**
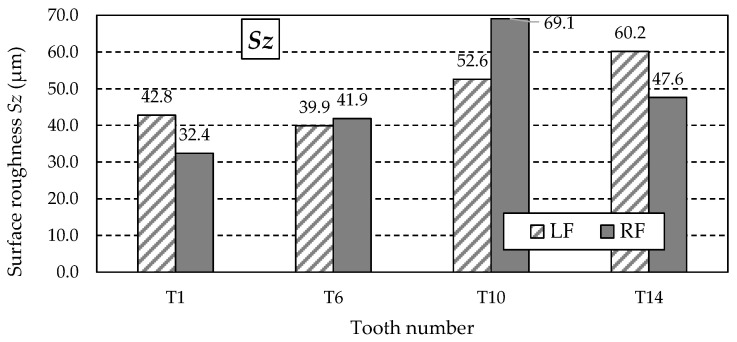
Values of the *Sz* parameter for the left flank (LF) and right flank (RF) of the measured teeth—after printing, before grinding.

**Figure 18:**
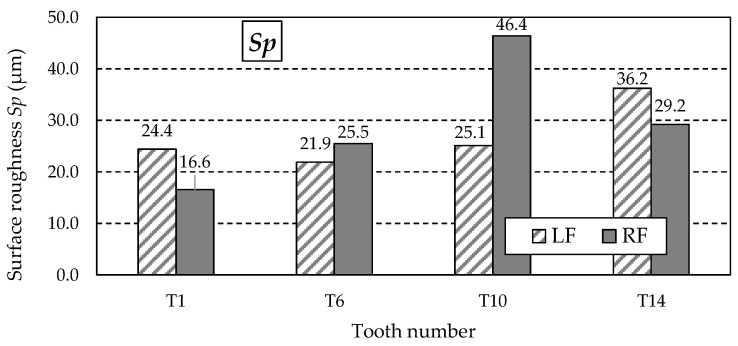
Values of the *Sp* parameter for the left flank (LF) and right flank (RF) of the measured teeth—after printing, before grinding.

The possibility of unusual valleys or peaks on the measured working surfaces may be indicated by the obtained *Rz* parameter values, a graph of which is shown in Figure 17. As can be observed, this mainly applies to the right flank (RF) of tooth T10 and the left flank (LF) of tooth T14, for which the *Rz* values are significantly higher than the others. The analysis of the *Sp* and *Sv* parameter values presented in Table 11 showed that their values are mainly determined by the highest peak of the *Sp* parameter (Figure 18).

**Figure 19:**
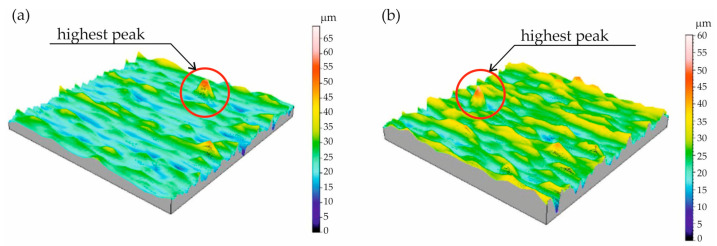
Single abnormal peak on: (**a**) right flank of T10 tooth; (**b**) left flank of T14 tooth.

The images shown in Figure 19 confirm the presence of an unusual single peak affecting the obtained *Sz* parameter value. A single unusually high peak that may influence the *Sz* and *Sp* parameter values is marked. The images represent the right flank of tooth T10 (Figure 19a) and the left flank of tooth T14 (Figure 19b).

**Figure 20:**
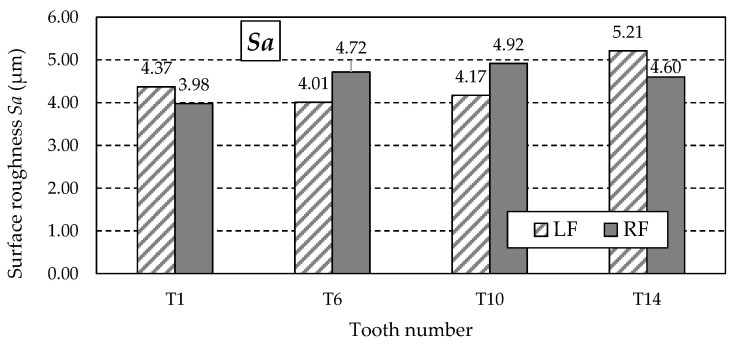
Values of the *Sa* parameter for the left flank (LF) and right flank (RF) of the measured teeth—after printing, before grinding.

Furthermore, upon analysing the *Sz* parameter values (Figure 17), it can be concluded that the roughness of the working surface of the gear teeth produced by the applied additive method is significantly (several times) more significant than the roughness obtained in the case of machining. Figure 20 shows the graph of the *Sa* parameter values, which is the arithmetic mean of the surface roughness deviation within the sampling area, and, therefore, its value is less dependent on the occurrence of a single peak, as is the case with the *Sz* parameter.

#### 3.4.2. Parameters of Tooth Working Surface After Grinding

**Figure 21:**
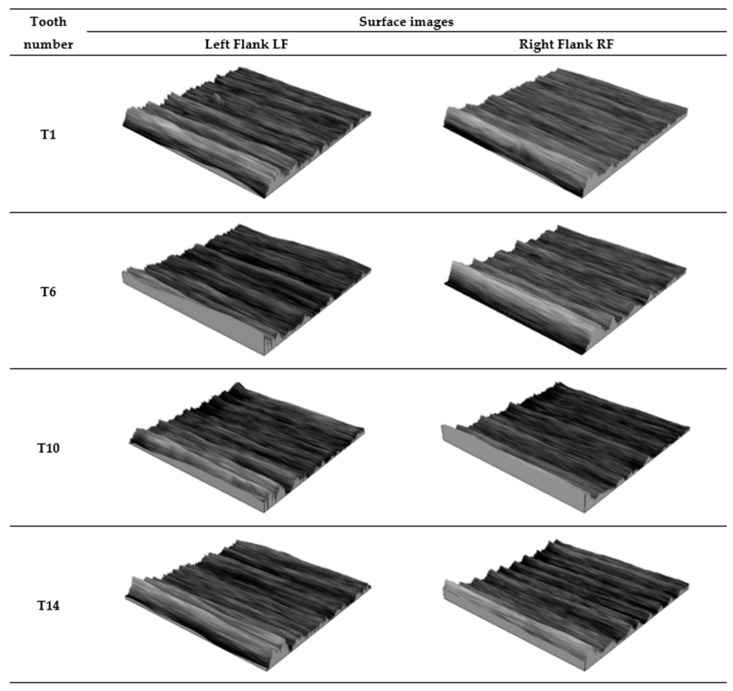
Images of the working surface of the measured teeth—after grinding.

**Figure 22:**
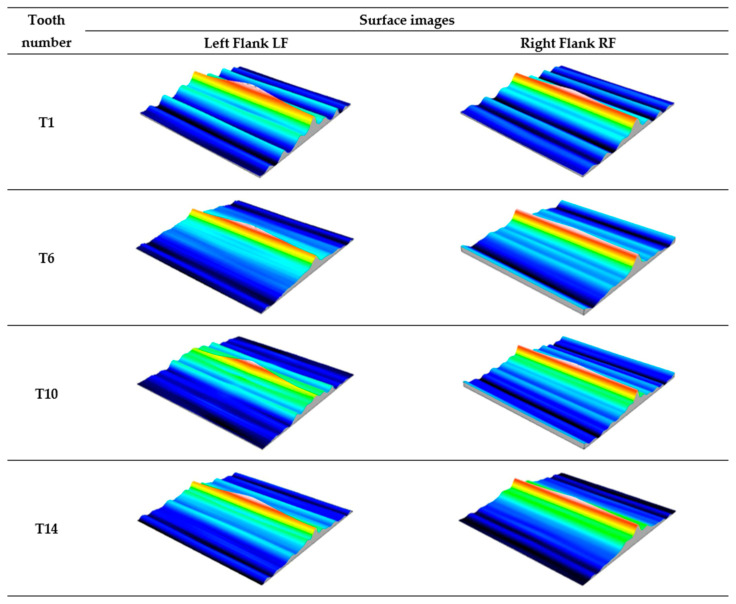
Autocorrelation functions of the working surface of the measured teeth—after grinding.

Figure 21 summarises the isometric views of the tooth working surface after grinding, while Figure 22 shows images of the autocorrelation function of the measured surfaces.

Based on the isometric images of the surfaces presented in Figure 21, it can be preliminarily observed that the working surface of all teeth after grinding, both on the left and right flanks, shows signs of an anisotropic surface. The directionality of the geometric surface structure is visible, resulting from the grooves formed due to the working contact of the abrasive grains of the grinding wheel with the material being processed during grinding.

The analysis of the autocorrelation function images of the measured surfaces shown in Figure 22 indicates the presence of a random anisotropic surface, typically obtained after abrasive processing. Similar to the surfaces directly after printing Figure 14, the images noticeably show the periodic components of a random nature, caused by disturbances in the technological process.

**Figure 23:**
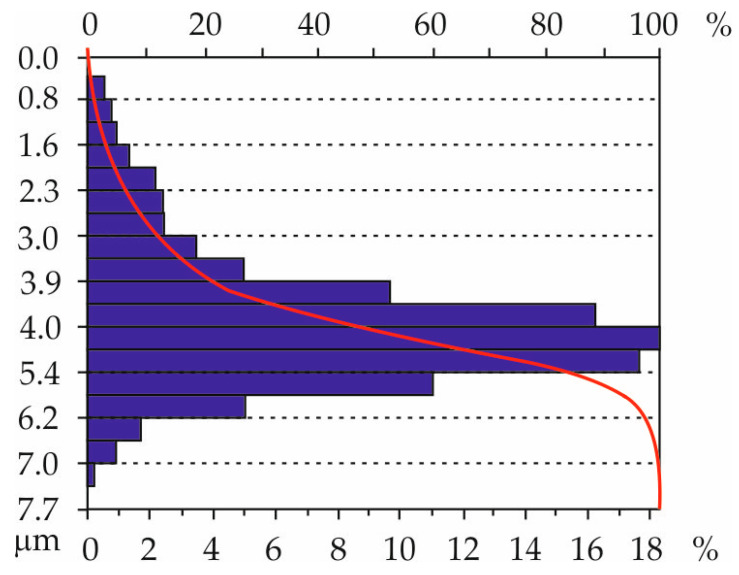
Example distribution of ordinates of the characteristic geometric structure of the surface after grinding; T6 tooth, right flank.

The obtained distributions of the ordinates of the characteristic geometric surface formations confirm the acquisition of random surfaces. As for such surfaces, the ordinate distribution approaches a normal distribution. Figure 23 shows an example graph of one of the surfaces (tooth T6, right flank).

The surface texture index *Str*, whose values are listed in Table 12, confirms the anisotropic nature of the examined surfaces. All obtained *Str* values are close to 0, characteristic of anisotropic surfaces.

The isotropy values listed in Table 12 confirm the anisotropy of the obtained surfaces. In all cases, the level of isotropy does not exceed 10%, indicating an anisotropic structure.

Table 13 lists the remaining 3D parameter values obtained from the measurements. These are amplitude parameters and include *Sz*, *Sa*, *St*, *Sp*, *Sv*, and *Ssk*.

**Figure 24:**
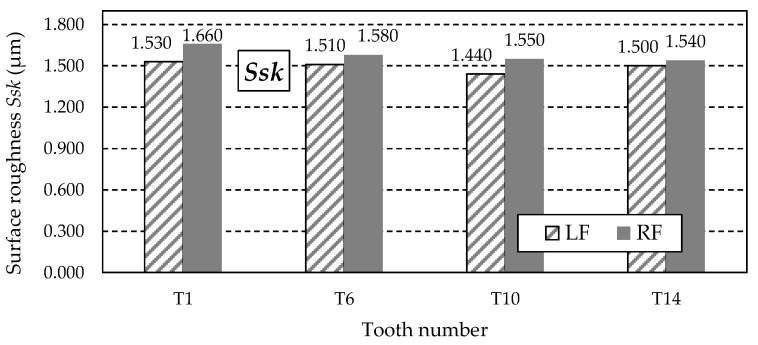
Skewness coefficient *Ssk* for the left flank (LF) and right flank (RF) of the measured teeth—after grinding.

Figure 24 shows the graph of the *Ssk* parameter values. The positive values of the *Ssk* parameter obtained from the measurements indicate surfaces with sharp peaks.

**Figure 25:**
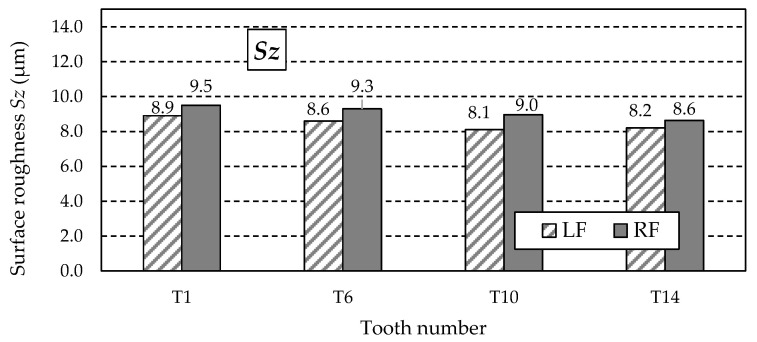
Values of the *Sz* parameter for the left flank (LF) and right flank (RF) of the measured teeth—after grinding.

**Figure 26:**
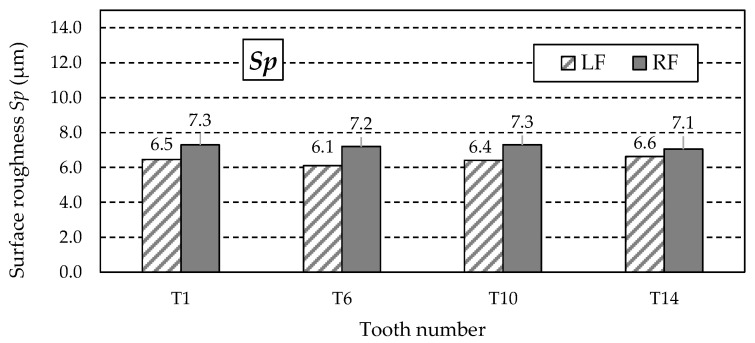
Values of the *Sp* parameter for the left flank (LF) and right flank (RF) of the measured teeth—after grinding.

The similar values of the *Sz* parameter (Figure 25) do not indicate the possibility of abnormal valleys or peaks on the measured working surfaces. This is confirmed by analysing the *Sp* and *Sv* parameter values listed in Table 13. The values of the *Sp* parameter (Figure 26) obtained for individual teeth are similar, as is the case with the *Sv* parameter.

**Figure 27:**
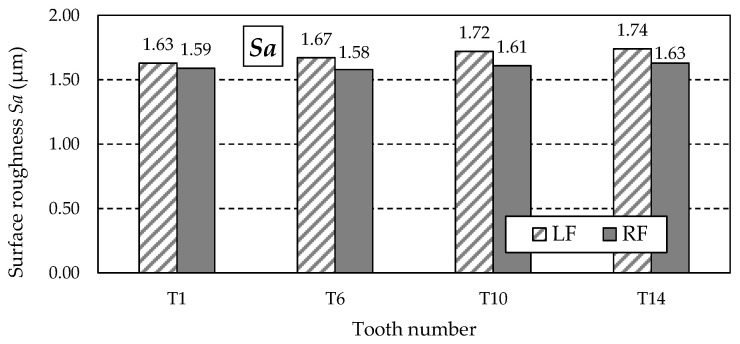
Values of the *Sa* parameter for the left flank (LF) and right flank (RF) of the measured teeth—after grinding.

Figure 27 shows the graph of the *Sa* parameter values, which is the arithmetic mean of the surface roughness deviation within the sampling area. For this reason, its value is less dependent on the occurrence of individual peaks, as is the case with the *Sz* parameter.

### 3.5. Results of Dimensional and Shape Measurements of the Teeth

#### 3.5.1. Radial Runout *Fr*

Table 14 lists the two values of the radial runout *F_r_* measured for the gear directly after printing and then after grinding the teeth’s working surfaces. The accuracy class of the gear teeth resulting from the obtained results is also included.

Based on the above results, it should be stated that the grinding operation of the gear teeth resulted in a favourable runout of the teeth, as well as a more than sevenfold reduction in the value of the radial. This led to a significant improvement in the geometry of the teeth, confirming the achievement of the third accuracy class.

#### 3.5.2. Tooth Thickness *s*

Table 15 lists the tooth thickness values *s* obtained from the measurements of the examined gear teeth. These include the minimum value *s_min_*, the maximum value *s_max_*, and the average value *s_ave_*. Additionally, the table consists of the values of the difference between the measured values and the nominal thickness *s_CAD_* = 4.380 mm, derived from the dimensions of the 3D CAD model of the gear.

**Figure 28:**
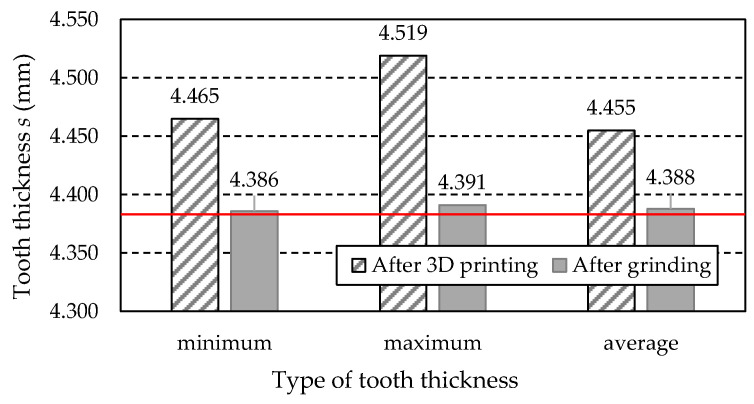
The measured values of tooth thickness s.

Figure 28 shows the graphical illustration of the data presented in Table 13. The level indicated by the nominal tooth thickness *s_CAD_* of 4.380 mm is marked with a red line.

Based on the measurement results presented above, the tooth thickness of the gear after printing increased compared to the nominal dimension derived from the 3D CAD model. The largest difference for a single tooth is 0.139 mm greater than the nominal value, while the smallest difference is 0.085 mm. The increase in tooth thickness dimensions is beneficial for their further grinding processing, as it removes excess material, improves the shape and line of the teeth, and reduces the roughness of their working surfaces.

#### 3.5.3. Tip Diameter *d_a_*

Table 16 lists the values of the tip diameter *d_a_* obtained from the measurements of the examined gear teeth. These include the minimum value *d_a min_*, the maximum value *d_a max_*, and the average value *d_a ave_*. Additionally, the table includes the values of the difference between the measured values and the nominal diameter *d_a CAD_* = 60 mm, derived from the dimensions of the gear’s 3D CAD model.

**Figure 29:**
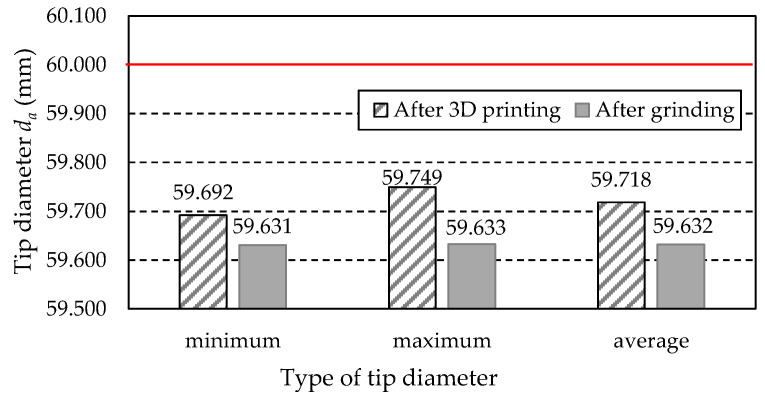
Measured values of the tip diameter *d_a_*.

A graphic illustration of the data in Table 14 is shown in Figure 29. The level indicated by the nominal tip diameter *d_a CAD_* of 60 mm is marked with a red line.

Based on the measurement results presented above, it can be stated that the tip diameter *d_a_* of the printed gear (before grinding) decreased compared to the nominal dimension derived from the 3D CAD model. Its smallest value is 59.692 mm, 0.308 mm less than the nominal value. It should be noted that the diameter of the outer cylindrical surface of gears produced using subtractive methods is usually not less than 0.01 to 0.02 mm compared to the nominal dimension. Therefore, in this case, the dimensional difference is significant.

#### 3.5.4. Profile Form Deviation *f_fα_*

Table 17 lists the values of the profile form deviation *f_f__α_* for the left and right flanks of the teeth (LF and RF) obtained from the measurements of four teeth distributed around the circumference of the gear at approximately 90° intervals, marked with symbols T1, T6, T10, and T14 (Figure 6). Based on the results, information about the accuracy class of the gear teeth is also included.

**Figure 30:**
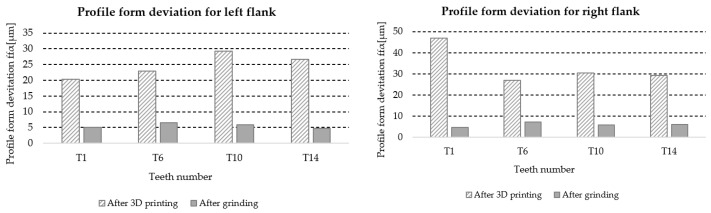
Graphic illustration of profile form deviation *f_fα_*.

A graphic illustration of the data in Table 17 is shown in Figure 30.

#### 3.5.5. Profile Slope Deviation *f_Hα_*

Table 18 lists the values of the profile slope deviation *f_Hα_* for the left and right flanks of the teeth (LF and RF) obtained from the measurements of four teeth distributed around the circumference of the gear at approximately 90° intervals, marked with symbols T1, T6, T10, and T14 (Figure 6). Based on the results, information about the accuracy class of the gear teeth is also included.

**Figure 31:**
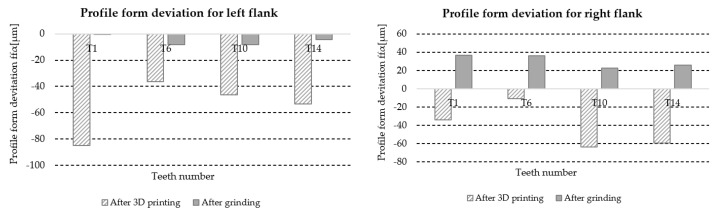
Graphic illustration profile form deviation *f_Hα_*.

A graphic illustration of the data in Table 18 is shown in Figure 31.

#### 3.5.6. Helix Form Deviation *f_fβ_*

Table 19 lists the values of the helix form deviation f_fβ_ for the left and right flanks of the teeth (LF and RF) obtained from the measurements of four teeth distributed around the circumference of the gear at approximately 90° intervals, marked with symbols T1, T6, T10, and T14 (Figure 6). Based on the results, information about the accuracy class of the gear teeth is also included.

**Figure 32:**
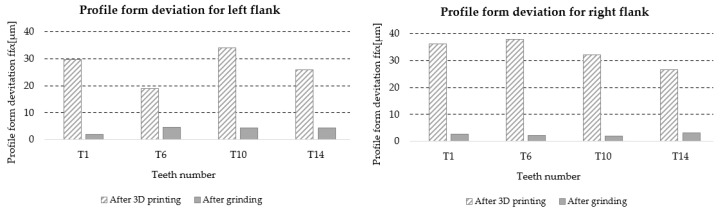
Graphic illustration of profile form deviation *f_f__β_*.

A graphic illustration of the data in Table 17 is shown in Figure 32.

#### 3.5.7. Helix Slope Deviation *f_Hβ_*

Table 18 lists the values of the helix slope deviation *f_H__β_* for the left and right flanks of the teeth (LF and RF) obtained from the measurements of four teeth distributed around the circumference of the gear at approximately 90° intervals, marked with symbols T1, T6, T10, and T14 (Figure 6). Based on the results, information about the accuracy class of the gear teeth is also included.

**Figure 33:**
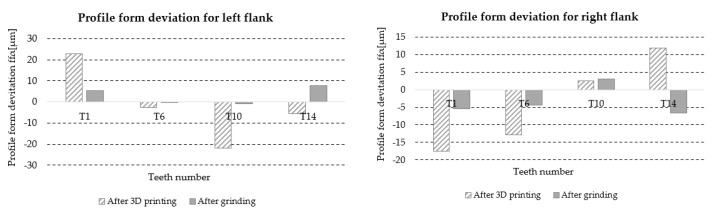
Graphic illustration of profile form deviation *f_H__β_*.

A graphic illustration of the data in Table 20 is shown in Figure 33.

### 3.6. Results of Central Hole Measurements

#### 3.6.1. Central Hole Diameter *D*

Table 21 lists the values of the hole diameter *D* obtained from measurements at three levels: L1, L2, and L3. Additionally, the table includes the values of the difference between the measured values and the nominal diameter *D_CAD_* = 30 mm, derived from the dimensions of the 3D CAD model of the gear.

**Figure 34:**
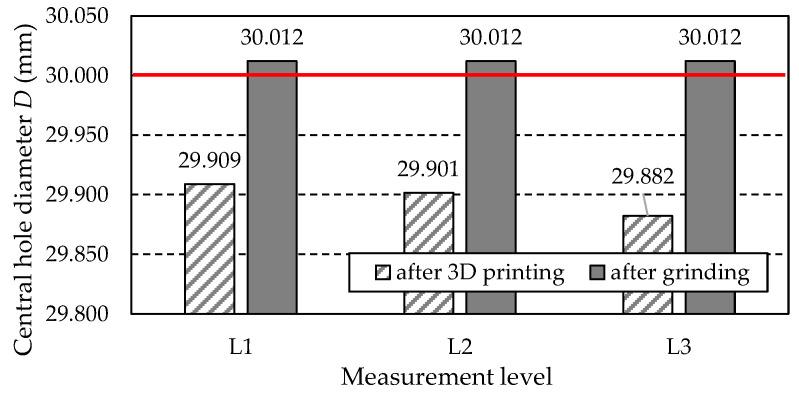
Measured values of central hole diameter *D*.

Figure 34 illustrates the data presented in Table 21 graphically. The nominal diameter *D* of 30 mm is marked with a red line.

**Figure 35:**
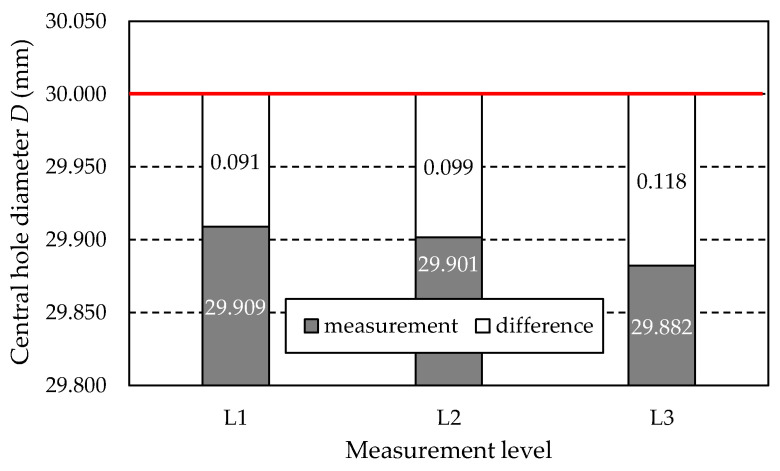
Measured values of central hole diameter *D* including allowances to be removed.

Figure 35 shows the graph of the hole diameter values along with information about the amount of excess material that needs to be removed to achieve the nominal diameter. The level indicated by the nominal hole diameter *D* of 30 mm is marked with a red line.

**Figure 36:**
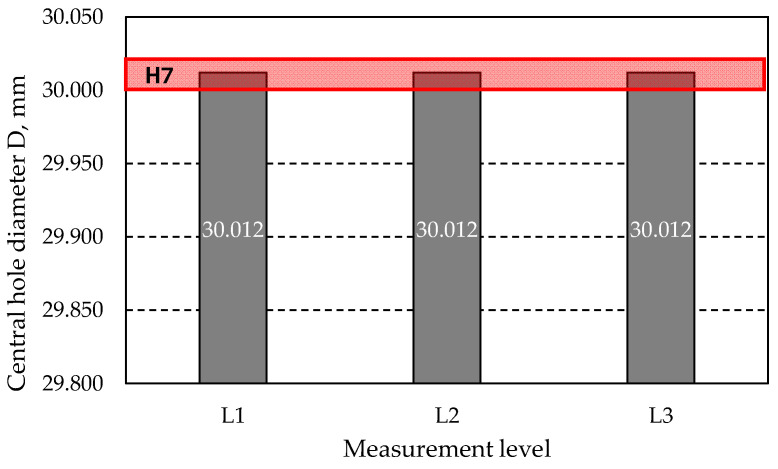
Values of central hole diameter *D* measured after grinding.

Figure 36 shows the graph of the hole diameter *D* at three measurement levels after the grinding process. The tolerance field for a hole with a nominal diameter of 30 mm is marked in red. As observed in the graph, the diameter at each level has the same value of 30.012 mm. The deviation of 0.012 mm classifies the obtained diameter within the H7 tolerance, for which the upper limit is 0.021 mm.

#### 3.6.2. Roundness

Table 22 summarises the roundness values obtained from measurements at three levels: L1, L2, and L3.

**Figure 37:**
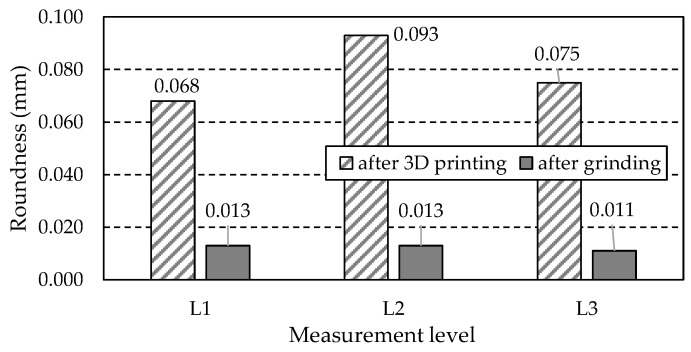
Central hole roundness obtained by measurement at levels L1, L2, and L3.

A graphic illustration of the data in Table 22 is shown in Figure 37.

#### 3.6.3. Cylindricity

Table 23 summarises the cylindricity values obtained from the measurements.

**Figure 38:**
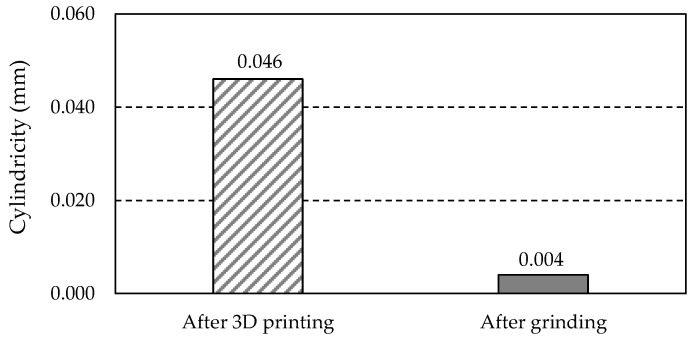
Measured values of central hole cylindricity.

A graphical illustration of the data in Table 23 is shown in Figure 38.

## 4. Discussion

Metallography plays a crucial role in analysing the structure of 3D printed materials, particularly following thermal processing. The additive manufacturing of metals, such as DMLS, is associated with the formation of characteristic artefacts in the microstructure, such as la-serial scan stripes. Heat treatment and other structure alignment techniques often improve mechanical properties and remove these artefacts. During DMLS, a laser beam scans the material in a specific pattern, creating layers. This process leads to the formation of characteristic scanning stripes that are visible in the metallographic images shown in Figure 9. The results of the EBSD analysis (Figure 11) show that, visually, the orientation of the grains does not possess a preferred crystallographic texture, as depicted by the colours of each grain.

The presented hardness results after heat treatment reached 54.3 HRC ± 0.6 HRC, similar to the results introduced in the article by H. Frank et al. [37]; therefore, it can be concluded that the material was subjected to effective heat treatment, which led to a hardness close to the maximum values possible for this steel alloy. Hardness at this level indicates the material’s good wear resistance and fatigue strength, which are crucial for components subjected to high contact and flexural loads.

The results of the stresses showed that compressive residual stresses could be found in most of the examined locations, which is a favourable phenomenon from the point of view of material strength. High compressive stress values were observed on the lateral surface of the tooth and at the bottom of the notch.

Since compressive stresses increase the material’s resistance to contact fatigue and bending, they are essential for improving the durability of components that operate under high cyclic loads [24].

The results indicate that the grinding process has drastically changed the stress state, leading to tensile stresses. Grinding generates local heating of the material, which leads to a thermal increase in stresses and the consequent formation of tensile stresses. Tensile stresses are disadvantageous because they increase the risk of micro-crack initiation and reduce the fatigue resistance of the surface.

During the study implementation, surface roughness parameters such as *Str*, *Sa*, *Sz*, *Sp*, *Sv*, and *Ssk* were determined for teeth with the following numbers: T1, T6, T10, and T14.

Knowing that the *Str* parameter describes how evenly the roughness features (e.g., grooves, peaks, valleys) are distributed in different directions on the surface, it is concluded that all of the tested teeth for both left and right flanks have anisotropic properties since all the results take values<1, i.e., the surface roughness structure is elongated or dominated by specific directions where pronounced grooves are formed. The anisotropic structure is important during friction and wear since anisotropic surfaces can have different friction properties in different directions, which has already been confirmed scientifically [38]. It is worth mentioning that adhesion is also significantly affected; for example, in the case of coatings or bonding, directionality can affect the quality of the joint. The literature mainly describes adhesion bonds between polymer materials from which models are made using 3D printing technology. However, one can find articles explaining the importance of adhesion during 3D printing from metals [39,40]. Each of the tested samples has similar values. The highest was recorded for the left flank of T1, which was 0.375, and the left flank of the T14 tooth had the lowest, which was 0.325.

Turning to the analysis of the other surface roughness parameters first, it should be noted that the highest values of the *Sz*, *Sp*, and *Sv* parameters were obtained for the T10 and T14 teeth. Knowing that *Sz* is the maximum height of the surface, *Sp* is the maximum height of the vertices, and *Sv* is the total height of the profile, it is noticeable that there are differences between the samples, which may indicate a variety of machining procedures or variable surface conditions. Samples T10 and T6 are characterised by the most significant values of *Sz* and *Sv*, suggesting that they may be of limited function in applications requiring smooth surfaces. Smooth surfaces, i.e., those with lower *Sa* and *Sz* values, will be more suitable in applications requiring low friction, such as mechanical systems. Surfaces with pronounced valleys can be advantageous in lubrication applications, where the valleys store grease, improving system life.

Differences are also noticeable between the left and right flanks in the same samples, suggesting some inconsistency in the machining process. To reduce these differences, the technological conditions must be verified.

Based on the gear teeth radial runout *F_r_* measurements presented in Table 14, it can be concluded that the grinding operation of the gear teeth resulted in a beneficial, more than sevenfold reduction in radial runout value. This led to a significant improvement in the teeth’s geometry and achieved a high-quality class of 3.

For the measurements of the gear teeth thickness after 3D printing, it should be noted that this dimension increased compared to the nominal dimension from the 3D CAD model. The most significant difference value is 0.139 mm greater than the nominal, while the smallest is 0.085 mm. It should be noted that the increase in teeth thickness is beneficial, as it allows for the removal of machining allowance through grinding. This, in turn, improves the profile and line of the teeth and reduces the roughness of the working surface of the gear teeth.

Based on the measured values of the tip diameters *d_a_*, it should be noted that the tip diameter of the printed gear (before grinding) decreased compared to the nominal dimension from the 3D CAD model. The smallest value is 59.692 mm, 0.308 mm less than the nominal dimension. It should be remembered that the diameter of the outer cylindrical surface of gears produced using machining methods is usually not less than 0.01 to 0.02 mm with the nominal dimension. Therefore, in this case, the dimensional difference is significant.

Based on the measurement results compiled in Table 21 and shown in Figure 34, the following can be stated in the case of the gear after printing (before grinding):

- At all three levels (L1, L2, L3), there was a reduction in the hole diameter compared to the nominal value resulting from the 3D CAD model of the gear.
- The most negligible reduction in the hole diameter was recorded in the L1 section, located closest to the gear wheel front surface, which, during printing, is furthest from the working platform of the printer table. In contrast, the largest reduction in diameter was found for the L3 section, located closest to the front surface, which, during printing, is closest to the working platform of the printer and is connected to the table by supports.
- For practical reasons, the most critical value is the largest measured hole diameter, as it determines the amount of material that must be removed in the machining process to achieve the nominal hole diameter.

Due to the last of the above points, Figure 35 shows a graph of the hole diameter values and information about the amount of material that must be removed to obtain the nominal diameter. As shown in Figure 35, the amount of material that needs to be removed to achieve the nominal hole dimension is limited by the diameter obtained at level L1, which is 29.909 mm. This means that the amount of material to be removed is 0.091 mm, which can be removed in the finishing process by grinding the hole to achieve a dimension within the tolerance, e.g., H7.

The measurement results presented in Figure 37 indicate that grinding significantly and beneficially reduced the hole’s roundness at each of the three measurement levels. For level L1, the roundness value after grinding is more than five times smaller than the hole’s roundness before grinding. Similarly, for level L2, the roundness is more than seven times smaller, and for level L3, it is almost seven times smaller.

It is worth noting that the grinding process also reduced the differences in roundness between the measured levels. This difference is only 0.002 mm. In the case of the hole’s roundness before grinding, the maximum difference between the roundness value obtained for level L1 and the roundness value obtained for level L2 is 0.025 mm, constituting almost 37% of the former’s value.

Based on the measurement results presented in Figure 38, grinding significantly and beneficially reduced the hole’s cylindricity. Its value after grinding is 11.5 times smaller than that obtained for the hole before grinding.

## 5. Conclusions

Based on the presented discussion, the following conclusions can be drawn:

- After heat treatment, clear traces of directionality and micropores are visible.
- Heat treatment increases the material’s hardness to 54.3 HRC ± 0.6 HRC, indicating effective strengthening.
- Compressive residual stresses are present in most examined areas, which is beneficial for material strength. Grinding changes the stress state to tensile, which is disadvantageous as it increases the risk of micro-crack initiation.
- All tested teeth exhibit anisotropic roughness properties, significant for friction and wear.
- Grinding the teeth significantly reduced radial runout, improving the gear geometry.
- After printing, the tooth thickness increased compared to the nominal dimension, which is advantageous for further grinding.
- The tip diameter decreased after printing compared to the nominal dimension.
- The central hole diameter decreased at all three levels (L1, L2, L3) after printing. Grinding significantly reduced the roundness and cylindricity of the holes, improving their accuracy.

## Figures and Tables

**Table 1:** Nominal gear wheel dimensions.

Parameter	Value
Module	*m* = 3 mm
Number of teeth	z = 18
Reference diameter	*d* = 54 mm
Tip diameter	*d_a_* = 60 mm
Facewidth	*b* = 10 mm
Pressure angle	*α* = 20°
Helix angle	*β* = 0°
Profile shift coefficient	*x* = 0
Central hole diameter	*D* = 30 mm
Tooth profile	Involute

**Table 2:** The chemical composition of 1.2709.

Content of Elements (wt.%)												
	Ni	Co	Mo	Ti	Al	Cr	Cu	C	Mn	Si	P	S
Min.	17	8.5	4.5	0.6	0.05	-	-	-	-	-	-	-
Max.	19	9.5	5.2	0.8	0.15	0.5	0.5	0.03	0.1	0.1	0.01	0.01

**Table 3:** The process parameters considered to prepare 3D-printed gears.

Parameter	Value
Layer thickness	40 μm
Scanning method	Stripe/Zigzag
Stripe width	10 mm
Overlap stripes	0.08 mm
Hatch distance	0.11 mm
Adjacent layers angle	67°
Building direction	the main gear axe
Laser powder	285 W
Laser speed	960 mm/s

**Table 4:** Cutting conditions used during flat surface grinding.

Grinding mode	Longitudinal circumferential flat surface grinding
Grinding machine	Flat-surface grinder SPD-30B made by Jotes Co., Ltd. (Łódź, Poland)
Grinding wheel	Vortex type—IPA60EH20VTX made by Norton Saint-Gobein Ltd. (Koło, Poland)
Grinding parameters	Grinding wheel peripheral speed vs. = 30.2 m/s
	Workpiece speed *v_w_* = 18 m/min
	Working engagement (depth of cut) *a_e_* = 0.01 mm
	Machining allowance *a* = 0.3 mm
Dressing parameters	Dresser type: single diamond dresser type M1020
	Dresser weight *Q_d_* = 2.0 kt (0.4 g)
	Axial table feed speed while dressing *v_fd_* = 5.0 m/s
	Grinding wheel peripheral speed while dressing *v_sd_* = 10 m/s
	Dressing allowance *a_d_* = 0.01 mm
	Dressing passes *i_d_* = 4
Coolant	Coolant mode: conventional flood method
	Coolant type: water–oil emulsion with Emulgol ES-12 oil (5%)
	Flow rate *Q* = 4 L/min

**Table 5:** Cutting conditions used during internal cylindrical grinding.

Grinding mode	Internal cylindrical grinding
Grinding machine	Internal cylindrical grinder SOH 10 from Jotes Co., Ltd. (Łódź, Poland)
Grinding wheel	99AF60K5VBE
Grinding parameters	Grinding wheel peripheral speed *v_s_* = 22.3 m/s
	Workpiece speed *v_w_* = 17 m/min
	Working engagement (depth of cut) *a_e_* = 0.02 mm
	Machining allowance *a* = 0.2 mm
	Axial table feed speed *v_fa_* = 0.2 m/s
Dressing parameters	Dresser type: single diamond dresser type M1020
	Dresser weight *Q_d_* = 2.0 kt (0.4 g)
	Axial table feed speed while dressing *v_fd_* = 5.0 mm/min
	Grinding wheel peripheral speed while dressing *v_sd_* = 10 m/s
	Dressing allowance *a_d_* = 0.01 mm
	Dressing passes *i_d_* = 4
Coolant	Coolant mode: conventional flood method
	Coolant type: water–oil emulsion with Emulgol ES-12 oil (5%)
	Flow rate *Q* = 3.5 L/min

**Table 6:** Heat treatment parameters.

Ageing Heat Treatment Parameters	Temperature [°C]	Time [h]
Heating	22–490	1.5
Age-hardening	490	6
Cooling	with furnace	

**Table 7:** Parameters of the grinding wheel used during the tests.

Parameter	Value
Grinding wheel type (shape)	Type 4, both sides with a tapered wheel (according to ISO 525)
Angle (face point to side)	20°
Symbol	99A80M8V
Abrasive grain	99A—white aluminium oxide
Bond type	V—vitrified bond
Hardness grade	M
Grain size	80
Structure	8—open
Grinding wheel dimensions *D* × *T* × *H*	340 × 20 × 127 mm

**Table 8:** Grinding parameters used during testing.

Parameter	Value
Grinding mode	Generating gear grinding using the Niles method
Grinding wheel rotational speed	*n_s_* = 1590 rev/min
Grinding wheel peripheral speed	*v_s_* = 28.3 m/s
Axial table speed	*v_st_* = 165 mm/min
Working engagement (machining allowance)	*a_e_* = 0.03 mm
Grinding wheel stroke length	*l_sk_* = 35 mm
Workpiece speed	*v_w_* = 10 m/min
Grinding wheel stroke frequency	*DH* = 100 double-stroke/min

**Table 9:** Surface roughness measuring conditions.

Parameter	Value
Type of profilometer	Hommel Tester T8000 made by Hommelwerke company (Schwenningen, Germany)
Stylus type	TKU 300
Tracing length	*lt* = 4.8 mm
Evaluation length	*ln* = 4.0 mm
Sampling length	*lr* = 0.8 mm
Evaluation width	*l* = 5 mm
Number of stylus passes	20
Distance between stylus tracks	0.25 mm
Stylus tip radius	*r_tip_* = 2 µm
Tracing speed	*v_t_* = 0.05 mm/s
Measuring range	±400 µm

**Table 10:** Texture aspect ratio and isotropy of measured surfaces.

Tooth Number	Tooth Flank	Texture Aspect Ratio *Str* (−)	Isotropy (%)
T1	LF	0.375	33.8
	RF	0.355	35.1
T2	LF	0.361	33.5
	RF	0.345	34.5
T3	LF	0.337	32.7
	RF	0.374	37.4
T4	LF	0.325	32.5
	RF	0.326	32.6

**Table 11:** Surface roughness 3D parameters—after printing, before grinding.

Tooth Number	Tooth Flank	*Sa* (μm)	*Sz* (μm)	*Sp* (μm)	*Sv* (μm)	*Ssk* (μm)
T1	LF	4.37	42.8	24.4	18.4	0.919
	RF	3.98	32.4	16.6	15.8	1.03
T6	LF	4.01	39.9	21.9	18	0.945
	RF	4.72	41.9	25.5	16.4	0.838
T10	LF	4.17	52.6	25.1	27.5	0.849
	RF	4.92	69.1	46.4	22.7	1.03
T14	LF	5.21	60.2	36.2	24	0.876
	RF	4.6	47.6	29.2	18.4	0.907

**Table 12:** Texture aspect ratio and isotropy of measured surfaces—after grinding.

Tooth Number	Tooth Flank	Texture Aspect Ratio *Str* (−)	Isotropy (%)
T1	LF	0.090	9.0
	RF	0.095	9.5
T6	LF	0.092	9.2
	RF	0.098	9.8
T10	LF	0.087	8.7
	RF	0.093	9.3
T14	LF	0.090	9.0
	RF	0.096	9.6

**Table 13:** Surface roughness 3D parameters—after grinding.

Tooth Number	Tooth Flank	*Sa* (μm)	*Sz* (μm)	*Sp* (μm)	*Sv* (μm)	*Ssk* (μm)
T1	LF	1.63	8.9	6.5	1.8	1.53
	RF	1.59	9.5	7.3	1.9	1.66
T6	LF	1.67	8.6	6.1	1.4	1.51
	RF	1.58	9.3	7.2	1.4	1.58
T10	LF	1.72	8.1	6.4	1.4	1.44
	RF	1.61	9.0	7.3	1.1	1.55
T14	LF	1.74	8.2	6.6	1.6	1.50
	RF	1.63	8.6	7.1	1.6	1.54

**Table 14:** Measured values of radial runout of teeth.

Gear Type	Radial Runout *F_r_* (μm)	Gear Tooth Quality
After 3D printing	74	7
After grinding	10	3

**Table 15:** Measured tooth thickness values.

Gear after 3D printing	Tooth thickness *s* (mm)		
	Minimum *s_min_*	Maximum *s_max_*	Average *s_ave_*
	4.465	4.519	4.455
	Difference between measured and nominal (*s_CAD_*) value		
	0.139	0.085	0.075
Gear after grinding	Tooth thickness *s* (mm)		
	Minimum *s_min_*	Maximum *s_max_*	Average *s_ave_*
	4.386	4.395	4.390
	Difference between measured and nominal (*s_CAD_*) value		
	0.110	0.006	0.008

**Table 16:** Measured values of the tip diameter.

Gear after 3D printing	Tip diameter *d_a_* (mm)		
	Minimum *d_a min_*	Maximum *d_a max_*	Average *d_a ave_*
	59.692	59.749	59.718
	Difference between measured and nominal (*d_a CAD_*) value		
	−0.308	−0.251	−0.282
Gear after grinding	Tip diameter *d_a_* (mm)		
	Minimum *d_a min_*	Maximum *d_a max_*	Average *d_a ave_*
	59.631	59.633	59.632
	Difference between measured and nominal (*d_a CAD_*) value		
	−0.369	−0.367	−0.368

**Table 17:** Measured values of profile form deviation *f_fα_* for the left and right flanks of the wheel teeth.

Gear Type	Teeth Number	Profile Form Deviation *f_fα_* (μm)			
		Left Flank LF	Gear Tooth Quality	Right Flank RF	Gear Tooth Quality
After 3D printing	T1	20.3	10	46.8	11
	T6	23.0		27.0	
	T10	29.3		30.4	
	T14	26.7		29.2	
After grinding	T1	5.0	6	4.7	6
	T6	6.5		7.3	
	T10	5.8		5.9	
	T14	4.8		6.0	

**Table 18:** Measured values of the profile slope deviation *f_Hα_* for the left and right flanks of the wheel teeth.

Gear Type	Teeth Number	Profile Slope Deviation *f_Hα_* (μm)			
		Left Flank LF	Gear Tooth Quality	Right Flank RF	Gear Tooth Quality
After 3D printing	T1	−85.0	13	−34.2	12
	T6	−36.4		−10.8	
	T10	−46.4		−63.8	
	T14	−53.3		−59.2	
After grinding	T1	−0.1	7	36.7	11
	T6	−8.1		35.8	
	T10	−8.2		22.5	
	T14	−4.5		25.9	

**Table 19:** Measured values of helix form deviation *f_fβ_* for the left and right flanks of the wheel teeth.

Gear Type	Teeth Number	Helixform Deviation *f_f__β_* (μm)			
		Left Flank LF	Gear Tooth Quality	Right flank RF	Gear Tooth Quality
After 3D printing	T1	29.6	10	36.1	10
	T6	18.9		37.8	
	T10	34.0		32.2	
	T14	25.9		26.5	
After grinding	T1	1.9	3	2.7	2
	T6	4.5		2.1	
	T10	4.2		2.0	
	T14	4.3		3.1	

**Table 20:** Measured values of the helix slope deviation *f_Hβ_* for the left and right flanks of the wheel teeth.

Gear Type	Teeth Number	Helixslope Deviation *f_H__β_* (μm)			
		Left Flank LF	Gear Tooth Quality	Right Flank RF	Gear Tooth Quality
After 3D printing	T1	22.9	9	−17.5	8
	T6	−2.5		−12.8	
	T10	−21.9		2.5	
	T14	−5.7		11.9	
After grinding	T1	5.4	6	−5.4	5
	T6	−0.1		−4.3	
	T10	−0.9		3.1	
	T14	7.6		−6.7	

**Table 21:** Measured values of the diameter of the central hole.

Gear Type	Level Number	Central Hole Diameter *D* (mm)	Difference Between Nominal and Measured Value (mm)
After 3D printing	L1	29.909	−0.091
	L2	29.901	−0.099
	L3	29.882	−0.118
After grinding	L1	30.012	0.012
	L2	30.012	0.012
	L3	30.012	0.012

**Table 22:** Central hole roundness obtained by measurement at levels L1, L2, and L3.

Gear Type	Level Number	Roundness (mm)
After 3D printing	L1	0.068
	L2	0.093
	L3	0.075
After grinding	L1	0.013
	L2	0.013
	L3	0.011

**Table 23:** Cylindricity.

Gear Type	Cylindricity (mm)
After 3D printing	0.046
After grinding	0.004

## Data Availability

The original contributions presented in this study are included in the article. Further inquiries can be directed to the corresponding authors.
